# Substantial blue carbon in overlooked Australian kelp forests

**DOI:** 10.1038/s41598-020-69258-7

**Published:** 2020-07-23

**Authors:** Karen Filbee-Dexter, Thomas Wernberg

**Affiliations:** 1 Institute of Marine Research, 4817 His, Norway; 2 UWA Oceans Institute, University of Western Australia, Crawley, WA 6009 Australia; 3 Department of Science and Environment, Roskilde University, 4000 Roskilde, Denmark

**Keywords:** Climate-change ecology, Marine biology

## Abstract

Recognition of the potential for vegetated coastal ecosystems to store and sequester carbon has led to their increasing inclusion into global carbon budgets and carbon offset schemes. However, kelp forests have been overlooked in evaluations of this ‘blue carbon’, which have been limited to tidal marshes, mangrove forests, and seagrass beds. We determined the continental-scale contribution to blue carbon from kelp forests in Australia using areal extent, biomass, and productivity measures from across the entire Great Southern Reef. We reveal that these kelp forests represent 10.3–22.7 Tg C and contribute 1.3–2.8 Tg C year^−1^ in sequestered production, amounting to more than 30% of total blue carbon stored and sequestered around the Australian continent, and ~ 3% of the total global blue carbon. We conclude that the omission of kelp forests from blue carbon assessments significantly underestimates the carbon storage and sequestration potential from vegetated coastal ecosystems globally.

## Introduction

The rapidly changing climate provides a strong impetus to uncover sinks in the global carbon cycle, in order to identify possible ways to mitigate current carbon emissions^1,2^. Vegetated coastal ecosystems store and sequester large amounts of organic carbon globally^3–5^, and this recognition has recently led to their recent inclusion into global carbon budgets and carbon offset schemes^6^. Current accounting for this ‘blue carbon’ is restricted to vegetation in accreting coastal ecosystems, such as tidal marshes, mangrove forests, and seagrass beds, which have high internal carbon burial rates and accumulate carbon in their soils and sediments. In contrast, non-accreting vegetated coastal ecosystems dominated by large habitat-forming seaweeds (‘kelp forests’^7^) are not considered to contribute blue carbon^6,8,9^. Nevertheless, these ecosystems have large aboveground biomass with high detritus export rates^10^ and therefore represent substantial carbon stocks that could sequester carbon through processes other than local burial^11^, such as burial of allochthonous detritus in deep sea sediments^12^.

Kelp forests are extensive ecosystems that dominate a narrow band along 26% of the world’s coasts^13,14^, and predominantly grow on hard or mixed sand and rock substrate with little potential for local carbon burial^6,8^. However, new evidence suggests that these ecosystems do indeed sequester carbon as important donors of allochthonous biomass in other ecosystems^15–17^. On average kelp forests export ~ 80% of their production^10^, much of which leaves the nearshore as detrital particles and dissolved organic material and enters into deep coastal areas (400 m depth)^16^, the continental shelf and continental slope (1,800 m depth)^18^, and—in some cases—eventually reaches the deep sea (up to 4,000 m depth and 4,800 km away from the nearest coast)^12,19^. Estimates suggest that seaweeds sequester 153 Tg C year^−1^ in the deep sea globally^8,19^.

Australia’s Great Southern Reef extends ~ 8,000 km around the southern coastline of the continent, where it forms an extensive and often overlooked vegetated coastal ecosystem dominated by kelp forests^20^. We assessed the continental-scale contribution to blue carbon from kelp forests in Australia using areal extent, biomass, and productivity measures for its dominant kelp, *Ecklonia radiata*. We reveal that these kelp forests account for more than 30% of total blue carbon stored and sequestered around the continent by tidal marshes, mangrove forests and seagrass beds as reported by Serrano et al.^21^, and ~ 3% of the total global blue carbon.

## Results and discussion

**Figure 1:**
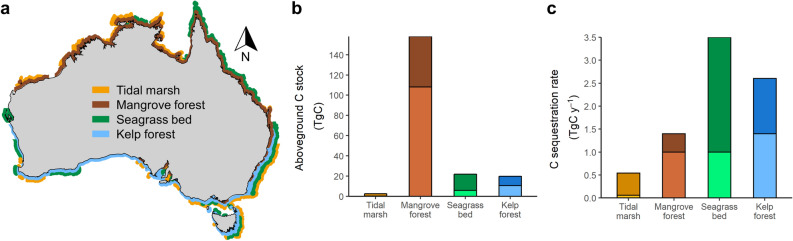
Kelp forest contribution to organic carbon standing stocks and sequestration rates for vegetated coastal ecosystems in Australia. (**a**) Spatial distribution of tidal marshes, mangrove forests, seagrass beds, and kelp forests. (**b**) Organic carbon stocks in aboveground biomass. (**c**) Sequestration rates across Australia. Stacked bars show maximum and minimum estimates. Data on tidal marshes, mangrove forests and seagrass beds are from Serrano et al. (2019). Data per unit area are provided in Table 1. The Map was generated in R using the mapdata package (A language and Environment for Statiscal Computing, R Core Team, R Foundation for Statiscal Computing, Vienna, Austria, 2017, https://www.R-project.org version 2.2–6, https://CRAN.R-project.org/package=mapdata), and ecosystems drawn in GIMP version 2.10.20 (https://www.gimp.org/.

We calculated that Australian kelp forests store an aboveground biomass of 10.3–22.7 Tg C and contribute 1.3–2.8 Tg C year^−1^ in sequestered production (see Supplementary Data). This represents 11–13% of the total standing stock of blue carbon and 27–34% of the annual blue carbon sequestration reported for the Australian continent (Fig. 1).

The total surface area of kelp forests in Australia is 3.2 to 7.1 Mha^20^. This is comparable to seagrass beds and 4 and 7 times higher than the extent of tidal marshes and mangrove forests, respectively (Table 1). Importantly, the distribution of kelp forests is largely disjunct from the other vegetated coastal ecosystems in Australia, with ~ 36% of kelp forests occurring in areas with no tidal marshes, mangrove forests or seagrass beds (Fig. 1a). This extensive ecosystem holds between 10 and 23 Tg C in aboveground biomass, which is similar to that of seagrass beds in Australia (Fig. 1b). We calculated that annual production per unit area of the dominant kelp species (*Ecklonia radiata*) on Australian reefs averages 3.9 Mg C ha^−1^ year^−1^ (± 0.9 SD) (Table 1). Based on the current best-estimate of proportion of net primary production (NPP) to become sequestered through burial in deep ocean sediments or transport below the mixed layer in the deep sea^19^, this represents an average sequestration rate per unit area of kelp forest of 0.39 Mg C ha^−1^ year^−1^ (± 0.09 SD). Although a coarse estimate, this rate is within the range of carbon sequestration per unit area of tidal marshes and seagrass beds and lower than mangrove forests, but when extrapolated over the total habitat area in Australia it forms a significant proportion (31%) of the total blue carbon sequestration rate (Fig. 1c). Indeed, our calculation may even underestimate the blue carbon contribution from Australian seaweed habitats substantially, as it does not include the extensive beds of *Sargassum* on tropical reefs in the north e.g.,^22^ or even more dominant fucoids^23^ and deep beds of red algae^24^ along the southern part of the continent, which, when combined with kelp forests, have been estimated to represent a total of 110 Tg C in aboveground biomass^11^.

**Table 1:** Blue carbon stocks (a) and sequestration rates (b) by vegetated coastal ecosystems in Australia. Estimates for tidal marshes, mangrove forests, and seagrass beds are from Serrano et al.^21^.

Ecosystem	Above ground biomass (Mg C ha^−1^)	Total area (Mha)	Stock above ground biomass (Tg C)
	Mean ± SD	Range	Range
(a)			
Tidal marshes	7.5 ± 6.1	1.4–1.5	2.3–2.6
Mangrove forests	125 ± 90	0.3–1.1	50–158
Seagrass beds	1.9 ± 2.0	9.3–12.8	16–22
Kelp forests	3.2 ± 0.5	3.2–7.1	10–23
Total		14.2–22.5	77–206

Ecosystem	Sequestration rates (Mg C ha^−1^ year^−1^)	Total area (Mha)	Sequestration rates (Tg C year^−1^)
(b)			
Tidal marshes	0.39 ± 0.3	1.4–1.5	0.48–0.54
Mangrove forests	12.6 ± 0.9	0.3–1.1	0.4–1.4
Seagrass beds	0.36 ± 0.3	9.3–12.8	2.5–3.5
Kelp forests	0.40 ± 0.1	3.5–7.1	1.3–2.8
Total		14.2–22.5	4.9–8.5

A key challenge of including kelp forests in blue carbon assessments is that kelp carbon may end up in, and be accounted for indirectly in, estimates from other blue carbon ecosystems, because significant amounts of seaweed detritus (i.e., epiphytic and drifting seaweed) can be buried in tidal marshes, mangrove forests and seagrass beds^5,25–27^. According to the estimate from Krause-Jensen and Duarte^19^, 11% [range = 4–18%] of all seaweed NPP is sequestered, and this percentage is almost entirely composed of NPP that reaches the deep ocean (> 1,000 m). Only 0.9% of NPP is buried on the entire continental shelf, such that an even much smaller proportion of this 0.9% would deposit in shelf habitats such as tidal marshes, mangrove forests and seagrass beds and be at risk of double counting. To ensure that kelp sequestration was not already accounted for as allochthonous seaweed derived carbon in estimates of carbon burial in other blue carbon systems (e.g.,^21^), our calculations conservatively excluded all burial on the continental shelf (0.9% of NPP) by using a sequestration rate of 10.1% NPP^19^. A more important challenge, however, is that the best estimates of the proportion of seaweed NPP sequestered in deep marine habitats are rudimentary. This represents a significant knowledge gap that must be closed to increase the confidence in estimates of kelp-derived blue carbon.

Conservation and restoration of blue carbon ecosystems are now being included in strategies to mitigate CO_2_ emissions^3,6^. There is current debate surrounding the application of these blue carbon strategies to coastal ecosystems other than tidal marshes, mangrove forests and seagrass beds^6,11,15^. Rooted vegetated marine ecosystems share commonalities with terrestrial ecosystems because they sequester carbon through *local* burial in accreting sediments, which is similar to carbon burial on land, such as in soil^28^. In contrast, accounting for carbon that is mainly sequestered as allochthonous detritus in the deep ocean^12,19^ is challenging for blue carbon policy because it is difficult to trace and to attribute a source to the site of storage, because of the risk of double-counting of material that ends up in other blue carbon ecosystems, and because sink habitats in the open ocean do not fall within national jurisdictions^6,8^. These are challenges for all blue carbon ecosystems, not only kelp forests. Export of detritus from tidal marshes, mangrove forests and seagrass beds is currently not considered to contribute to carbon sequestration, although detrital production from these habitats is likely substantial^29^. At the same time, the inability to trace allochthonous sources of buried carbon within tidal marshes, mangrove forests and seagrass beds currently prevents both accurate blue carbon accounting and allocations of carbon offset credits under many frameworks^6,30^. Regardless of the pervasive practical challenges around accounting for allochthonous carbon, kelp forests constitute important standing stocks of organic carbon and key components of organic carbon cycling in the coastal zone. Policy barriers and existing frameworks should not preclude their inclusion in our attempts to understand, quantify and manage carbon sources and sinks in the ocean.

Like most other blue carbon ecosystems, kelp forests follow a global trend of deterioration and decline, which is projected to worsen in the coming decades^7^. Australian kelp forests have been some of the worst impacted by human activities globally, and most regions of the Great Southern Reef have experienced kelp declines over the past decades^31^. Australia-wide ~ 1,000 km of coastline has been affected by kelp loss, totaling at least 140,187 ha (Table 2). Drivers of loss include an extreme marine heatwave^32^, coastal pollution^33,34^, warming and drought^35^, sea urchin overgrazing from climate-driven changes in the Eastern Australia Current^36,37^, and the influx of tropical herbivores with warmer waters^38^. In total these declines represent 0.45 Tg C of lost standing stock and 0.06 Tg C of lost annual sequestration. Importantly, these recorded losses come from reefs in intensively researched areas, and it is possible that similar declines have occurred throughout less studied regions of this remote ecosystem.

**Table 2:** Consequences of past (a) and future (b) losses of kelp forests in Australia on carbon standing stock and sequestration rates.

Region	Period	Driver	Coastline (km)	Cover loss (%)	Area loss (ha)	Carbon stock loss (Mg C)	Sequestration rate loss (Mg C year^−1^)
(a)							
Western Australia^1^	2005–2015	Marine heatwave	800	43.0	97,438	310,949	38,242
South Australia^2^	1968–2007	Coastal pollution	20	60	6,179	19,720	2,425
Victoria^3^	1958–2014	Warming and drought	40	85.8	17,665	56,375	6,933
Tasmania^4^	2001–2017	Sea urchin grazing	80	11.8	4,861	15,513	1,908
New South Wales^5^	2002–2010	Tropical fish grazing	25	88.7	11,414	36,425	4,480
Australia (total)			965		140,187	447,371	55,020

Region	Projections	Driver	Coast-line (km)	Cover loss (%)	Area loss (ha)	Carbon stock loss (Tg C)	Sequestration rate loss (Tg C year^−1^)
(b)							
Australia^6^	2100	Warming (RCP2.6)	8,000	49	34,981	8.1	1.0
Australia^6^	2100	Warming (RCP6.0)	8,000	71	50,686	11.8	1.4

^1^Wernberg et al.^32^, ^2^Connell et al.^33^, ^3^Carnell and Keough^35^, ^4^Ling and Keane^37^, ^5^ Vergés et al.^38^, ^6^Martínez et al.^39^. Calculations are provided in the Supplementary Data.

When kelp forests are lost, most of their carbon (89%)^19^ is incorporated into marine food webs and eventually remineralised as CO_2_, which can enter the atmosphere. As a result, potential changes in kelp forest area have important ramifications for carbon accounting strategies and predictions of carbon stocks in coming decades. By 2100 Australia’s *Ecklonia radiata* kelp forests are predicted to lose 49 to 71% of their current distribution under the RCP 2.6 and RCP 6.0 CO_2_ emission scenarios, respectively^39^. Even under the most optimistic scenario (RCP 2.6), this implies a loss of 6% of total blue carbon stock and a 15% loss of blue carbon sequestration rates for Australian vegetated coastal ecosystems (Table 2). Kelp forest management and restoration programs show potential to revert this alarming trajectory^40–42^. Restoration and proactive management actions could help minimize increased CO_2_ emissions from loss of standing stock and maintain valuable carbon sequestration rates from kelp forests, including along Australia's Great Southrn Reef. In order to scale up these national estimates to a global level, higher quality data on the areal extent and standing stock, as well as production, export and burial rates of kelps, such as those that exist for Australian kelp forests, are needed. Comprehensive and accurate estimates of blue carbon at large scales are critical for the success of blue carbon mitigation strategies and must include kelp forests if they are to fully capture the intense carbon storage and sequestration potential of the coastal zone.

## Methods

Kelp forest area was determined from suitable reef area and bounded by a lower depth limit of 30 m^20^. This represents a conservative estimate because kelps are often found to 45 m depth or more in several places along the Great Southern Reef^43^. The minimum and maximum extents were calculated by multiplying suitable reef area by the average percent cover ± SD of kelp (*Ecklonia radiata*) on 36 reefs across western Australia, southern Australia and eastern Australia, reported by Connell and Irving^44^. Carbon stock in Australian kelp forests were compiled from data collected across the full length of the Great Southern Reef; individual biomass data were obtained from 135 plants collected around Perth (3 sites, 15 plots), Adelaide (3 sites, 15 plots) and Sydney (3 sites, 15 plots)^45^ and from density data collected from 558 plots spread across New South Wales, Tasmania, South Australia and Western Australia (3 locations × 3 sites × 5–6 quadrats in each state) (Wernberg unpublished data). We calculated net primary productivity using 1,577 individual plant growth rates^31^ and 558 plots of kelp densities from across Australia. Carbon content in kelp tissue was assumed to be 30% of dry weight^46^. Carbon production rates were calculated using average net primary production measured from 7 separate tagging field studies across 7° longitude of coast^31^. We compared the contribution of kelp forests to other blue carbon habitats in Australia using data from Serrano et al.^21^.

We calculated historic blue carbon loss using estimates of the areal extent of kelp loss along the Great Southern Reef^32,33,35,37,38^. For time series data we averaged kelp abundance from the first 3 records and last 3 records (where available, see Supplementary Data). We also estimated future losses using the areal extent of range shifts modeled for the Great Southern Reef under different CO_2_ emission scenarios^39^. For studies not reporting the actual area of reef lost, we estimated reef area from coastline length using the average coastline to reef ratio reported from other regions of the Great Southern Reef^20,32^. We calculated the impacts of these events on standing stock and sequestration rates using average per area estimates for the entire Great Southern Reef (Table 1). Source data and calculations are provided as a Supplementary Data file.

## Supplementary information

Supplementary information

## Data Availability

A Supplementary Data file containing the raw data and calculations presented in the figures and tables is provided. Additional information can be obtained from the authors.
