# Author Correction: Substantial blue carbon in overlooked Australian kelp forests

**DOI:** 10.1038/s41598-020-74313-4

**Published:** 2020-10-09

**Authors:** Karen Filbee-Dexter, Thomas Wernberg

**Affiliations:** 1 Institute of Marine Research, 4817 His, Norway; 2 UWA Oceans Institute, University of Western Australia, Crawley, WA 6009 Australia; 3 Department of Science and Environment, Roskilde University, 4000 Roskilde, Denmark

Correction to: *Scientific Reports* https://doi.org/10.1038/s41598-020-69258-7, published online 23 July 2020

This Article contains an error in Figure 1b and c, where the bars for minimum and maximum standing carbon stock and sequestration rate have been flipped vertically. The correct data are reported in Table 1 and the corrected Figure appears below as Figure 1.

**Figure 1:**
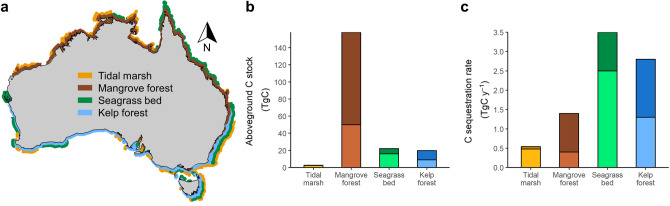
Kelp forest contribution to organic carbon standing stocks and sequestration rates for vegetated coastal ecosystems in Australia. (**a**) Spatial distribution of tidal marshes, mangrove forests, seagrass beds, and kelp forests. (**b**) Organic carbon stocks in aboveground biomass. (**c**) Sequestration rates across Australia. Stacked bars show maximum and minimum estimates. Data on tidal marshes, mangrove forests and seagrass beds are from Serrano et al. (2019). Data per unit area are provided in Table 1. The Map was generated in R using the mapdata package (A language and Environment for Statiscal Computing, R Core Team, R Foundation for Statiscal Computing, Vienna, Austria, 2017, https://www.R-project.org version 2.2–6, https://CRAN.R-project.org/package=mapdata), and ecosystems drawn in GIMP version 2.10.20 (https://www.gimp.org/).

